# Supplementary material for: Classification of multicategory edible fungi based on the infrared spectra of caps and stalks
Source: PLoS One. 2020 Aug 24;15(8):e0238149. doi: 10.1371/journal.pone.0238149 (PMC7444812; doi:10.1371/journal.pone.0238149)
Supplement: Attachment — Submitted filename: Response to Reviewers.doc [file pone.0238149.s002.doc]

Subject: Revised version of the manuscript

No.: PONE-D-20-12067

Title: **Classification of Multicategory Edible Fungi based on the Infrared Spectra of Caps and Stalks**

Dear Editor and Reviewer:

Thanks to the editor and reviewer for your patient review and important suggestion for our article. We have further revised your valuable suggestions. We have highlighted our manuscript changes through the use of **red font** in the revised manuscript.

The revision was carried out as a result of the comments of the editor and the reviewer.

The main corrections in the paper and the responds to the editor’s comments and the responds to the reviewer’s comments are as following:

**Editor :**

**Response to comment (1):**

( We understand that you purchased mushrooms from local markets for this study. In your Methods section, please provide additional details regarding the source of this material. Please provide the geographic coordinates and names of the purchase locations (e.g., stores, markets), if available, as well as any further details about the purchased items (e.g., lot number, source origin, description of appearance) to ensure reproducibility of the analyses.)

**Response-1:** Many thanks to the editor for the suggestions. Based on your suggestions, we have supplemented the description of the source origin, names of the purchase locations and appearance of mushrooms in **2. Experimental methods** to ensure reproducibility of the analyses. Thanks again to the editor for your valuable suggestions.

The contents are as follows:

Among them, *Lentinus edodes* are produced in Fujian Province of China, and purchased from Fuchang Food Limited Company, Fujian Province of China; *club fungi* are produced in Yunnan Province of China, purchased from Wuweijin Store, and *Bachu mushrooms* are produced in Bachu County, Xinjiang Province, and purchased from the most famous wholesale market in Urumqi -- Six Markets. The three kinds of mushrooms purchased are all dried. The caps of the three kinds of mushrooms are umbrella-shaped and dark brown. The stalks of *club fungi* are longer and those of *Lentinus edodes* and *Bachu mushrooms* are shorter. The three kinds of mushrooms are similar in appearance. The samples of the three types of edible fungi were purchased from the market.

**Reviewer #1:**

1. **Response to comment (1-4):**

**Response:** Thank you very much for the reviewer's agreement on our work, which will encourage us to do better work in the future.

2. **Response to comment (5):**

( The manuscript is very well written all the statistical analysis have been done with great rigour and very nicely presented. The manuscript is very easy to follow and targeted for chemometricians. My only comments is to specify which version of Matlab was used and to italicise the biological genus and species names of the fungi. Good job once again to all the authors involved in this publication.)

**Response-5:** Thanks to the reviewer for the suggestions. We have supplemented the MATLAB version and italicised the biological genus and species names of the fungi in the article.

Thank the reviewer and editor for your patient and careful review. I hope you have good health and success in your work.

Sincerely yours,

Dr. Xiaoyi Lv, Dr. Cheng Chen
